# Rumination-Focused Cognitive-Behavioral Therapy for Chronic Low Back Pain: A Randomized Controlled Trial

**DOI:** 10.31661/gmj.v9i0.1722

**Published:** 2020-12-28

**Authors:** Ali Soleymani, Abbas Masjedi Arani, Seyed Ahmad Raeissadat, Mohammad Hassan Davazdahemami

**Affiliations:** ^1^Department of Clinical Psychology, Taleghani Hospital, Shahid Beheshti University of Medical Sciences, Tehran, Iran; ^2^Department of Physical Medicine and Rehabilitation, Modarres Hospital, Shahid Beheshti University of Medical Sciences, Tehran, Iran

**Keywords:** Rumination, Low Back Pain, Depression, Anxiety

## Abstract

**Background:**

Chronic pain remains or reappears for more than 3 to 6 months, and it is influencing 20% of the global population. The pain catastrophizing affects pain intensity and psychological conditions of patients with chronic pain. Rumination-focused cognitive-behavioral therapy (RFCBT) targets rumination as the key component of pain catastrophizing. The aim of this study was to determine the effectiveness of RFCBT on depression, anxiety, and pain severity of individuals with chronic low back pain (LBP).

**Materials and Methods:**

In a randomized controlled trial, 30 patients aged between 20-55 years with diagnosed chronic LBP were chosen by convenience sampling and randomly allocated into intervention and control groups. All patients used their prescribed medications for pain management, but the intervention group received 12 weekly sessions of RFCBT, which was manualized psychotherapy to change unconstructive rumination to constructive rumination. Depression Anxiety and Stress scale-21 and chronic pain grade questionnaire were administered as pre-tests and re-administered after 3 and 6 months as post-test and follow-up assessments, respectively.

**Results:**

RFCBT significantly reduced depression (F1=23.01, P=0.001), anxiety (F1=25.7, P=0.001) and pain severity (F1=7.17, P=0.012) in patients with chronic LBP.

**Conclusion:**

RFCBT may offer benefits for treating patients with chronic low back pain when added to their usual pharmacological treatment. This benefit may be the result of targeting rumination as the key element of pain catastrophizing.

## Introduction

The worldwide literature usually depicts pain as a symptom of modifiable disease, injury or trauma, and its presence is assumed to mirror a failure of worldwide health activities to address a fundamental cause [1]. Usually, pain is viewed as chronic when it remains or reappears for more than 3 to 6 months [2]. The more elevated psychological disorders, especially depression and anxiety, are diagnosed in patients with chronic pain [3]. Low back pain (LBP) is defined as pain, tension, or rigidity localized below the costal margin and above the inferior gluteal folds with or without the radicular syndrome, which can be diagnosed in 70–85% of people at some moment in their lives in developed countries as a growing public health problem [4]. Almost 20% of LBP patients turn into chronic LBP (CLBP) with constant symptoms at one year. Studies showed that most of the patients with CLBP are diagnosed with depression and anxiety and experience higher pain severity and pain-related disability, leading to economic burden and low quality of life [4]. Negative psychological results of chronic pain showed that it is beneficial to consider three psychological mechanisms that are linked with pain-related stress and reasonable targets for psychological interventions; pain catastrophizing, fear of pain, and pain acceptance [5]. Pain catastrophizing is portrayed by amplifying the negative impacts of pain, rumination about pain, and feeling hopelessness in coping with pain. Pain-related fear mirrors a fear of injury or worsening one’s physical condition through activities that may trigger pain, and pain acceptance is characterized as a procedure of nonjudgmentally acknowledging the pain, stopping maladaptive endeavors to control pain, and figuring out how to live a better life regardless of the pain. Different researches have shown that these psychological mechanisms are associated with depression, anxiety, functional impairment, and disability due to pain and quality of life [5]. Pain catastrophizing is more studied than other psychological mechanisms, and it is shown to be associated with greater pain intensity, disability, and psychological distress [6]. Pain catastrophizing, perhaps, is the most precise predictor of the treatment outcome for pain [7]. Psychological treatments for chronic pain are most likely to produce clinically significant benefits when targeting pain catastrophizing [8]. This construct has three factors, including rumination, magnification, helplessness in which rumination can be the most important one according to the factor analysis of the test, which measures pain catastrophizing [9].

Furthermore, rumination can predict disability [10] and is a contributing factor to pain intensity in CLBP [11]. Therefore, studying the effectiveness of the potential treatments for chronic pain that target rumination as the key factor of pain catastrophizing can be important and helpful. In contrast, the association between rumination and treatment outcome in CLBP is illustrated in other researches [12]. The relationship between rumination and impaired somatic health seems to be mediated by two general factors; it can magnify perceived symptoms (intensifying the pain) and cause somatic distress through several physiological pathways [12]. Rumination-focused cognitive–behavioral therapy (RFCBT) modifies cognitive–behavioral therapy to focus on rumination [13]. A few previous studies showed the effectiveness of RFCBT for residual depression [14] compared with treatment as usual [13]. Group RFCBT is more effective than group cognitive–behavioral therapy when adding to usual medical care [14], and web-based RFCBT effectively reduces stress and prevents depression [15]. The effectiveness of this therapy for chronic pain and comorbid depression and anxiety has not been determined in previous studies despite its therapeutic potential regarding the focus on rumination as a key element in pain management. The objective of this study was to determine the effectiveness of this intervention on depression, anxiety, and pain severity among individuals with CLBP.

## Materials and Methods

###  Sample Size Calculation

 To detect the difference between RFCBT and control groups with a two-tailed α of 0.05 and a (1-β) of 0.80, we needed 15 patients in each group. We allocated 20 patients in each group, considering 25%-30% anticipated dropout.

###  Participants

 In a randomized controlled trial with parallel groups, 40 patients with diagnosed CLBP who referred to physical medicine and rehabilitation clinic at Modares hospital in Tehran were selected by convenience sampling. Inclusion criteria were 20 to 55 years of age, reading and writing level of grade 6 at least, reaching minimum scores to be diagnosed with mild depression and mild anxiety in depression anxiety stress scale-21 (DASS-21; scores14 and 10, respectively). Also, participants with a history of bipolar disorders or psychotic disorders, substance dependence or substance-related disorders, and receiving any other psychotherapy during the study were excluded. All patients signed a written informed consent before participation.

###  DASS-21

This scale contains three self-report subscales with 21 items and measures the emotional state of depression, anxiety, and stress. The severity rating includes normal, mild, moderate, severe, and very severe, and seven questions are dedicated to each subscale. Scores of each subscale need to be multiplied by two, and subscales may range between 0 and 42 [16]. Cronbach alpha of depression, anxiety, and stress for this questionnaire are 0.97, 0.92 and 0.95, respectively [17].

###  Chronic Pain Grade Questionnaire (CPG)

This seven-item questionnaire provides a score to categorize patients with chronic pain into four classes according to the severity of their pain and the level of interference with daily life [18]. All items are scored on an 11-point Likert scale, with responses ranging from 0–10. Scores are calculated for three subscales: the characteristic pain intensity score, the disability score, and the disability points score [19]. The Cronbach’s alpha of CPG is more than 0.90, and the validity of this questionnaire was confirmed by factor analysis [20]

###  Interventions

RFCBT was consisted of up to 12 sessions lasting 60 min scheduled weekly or fortnightly (Table-1). This therapy is based on the idea that rumination can be helpful or unhelpful. The goal of this treatment is redirecting the patient from unhelpful to the helpful cognitive process. This therapeutic goal can be achieved by analyzing the extent of helpful or unhelpful rumination, detecting the associated behavior with each form of cognitive process and using alternative response behaviors as counter-ruminative responses. The identification of early warning signs of rumination and action plans for interruption is another important component of RFCBT [12]. In RFCBT, rumination is conceptualized as a type of avoidance, and functional analysis is utilized to encourage useful approach behaviors. RFCBT also utilizes functional analysis to understand that their rumination about negative self-experience can be helpful or unhelpful and guide them in changing their thinking style.

Furthermore, patients utilize guided imagery to reproduce past mental states like memories of being fully absorbed in an activity (for example, ‘flow’ or ‘peak’ experiences), which works counter to rumination [13]. Special treatment components were used based on assessment, functional analysis and formulation of each patient, but the first session of RFCBT was usually focused on assessment, familiarizing to treatment, and presenting some active interventions like relaxation, listening to a recording of the session and reading educational booklets. The later sessions are focused on replacing avoidance with the approach, problem-solving and coaching to identify and change the thinking style. The final sessions stabilize these changes, deal with the end of the treatment and make a plan like relapse prevention [21]. In our study, RFCBT was delivered by a Ph.D. candidate in clinical psychology who was supervised by two licensed Ph.D. clinical psychologists.

###  Data Collection

 All participants answered DASS-21 and CPG questionnaires. They were randomly allocated to RFCBT or control groups (20 patients in each group) after pre-treatment assessment. A computer-generated list of random numbers was used for the allocation of the participants. We assigned participants into RFCBT and control groups by simple randomization. Sequentially numbered opaque, sealed envelopes were used for allocation concealment. To prevent subversion of the allocation sequence, the name, date of birth, and national ID number of the participants were written on the envelope. Carbon paper inside the envelope transferred the information onto the allocation card inside the envelope. The envelopes were opened only after the enrolled participants completed all baseline assessments, and it was time to allocate the intervention. The admitting clerk was in charge of enrollment, and two independent medical residents in the physical medicine and rehabilitation clinic generated the random allocation sequence and assigned participants to interventions. All participants in both groups continued to use their prescribed medications for pain management during the study, but the intervention group received 12 sessions of RFCBT. Patients in both RFCBT and control groups conditions administered all questionnaires and scales after the last session of the RFCBT, and the follow-up was done three months later. At the end of the study, patients of the control group received 12 weekly sessions of RFCBT.

###  Study Outcomes

 The primary outcome was a reduction in depression, anxiety, and pain severity in patients with CLBP after receiving RFCBT for 12 weeks.

###  Ethical Standards

 All procedures performed in the present study involving human participants were following the ethical standards of the institutional and/or national research committee and with the 1964 Helsinki Declaration and its later amendments or comparable ethical standards. Informed consent was obtained from all individual participants included in the study. This trial was registered at the Iranian Registry of Clinical Trials (number: IRCT20190710044169N1), and was approved by the Research Ethics Committee of this university (approval code: IR.sbmu.MST.REC.1396.433).

###  Data Analysis

 Data were analyzed using SPSS 24(Armonk, NY: IBM Corp, USA). Mixed ANOVA was used to determine the effect of RFCBT on depression, anxiety, and pain intensity of participants. Normality, homogeneity of variance, and sphericity of the covariance matrix as ANOVA assumptions were tested before using mixed ANOVA to analyze data. Bonferroni was used as a posthoc test. Multivariate analysis of covariance was also used to analyze subscales of CPG, which showed a significant difference between RFCBT and control groups in at least one of the subscales.

## Results

**Figure 1:**
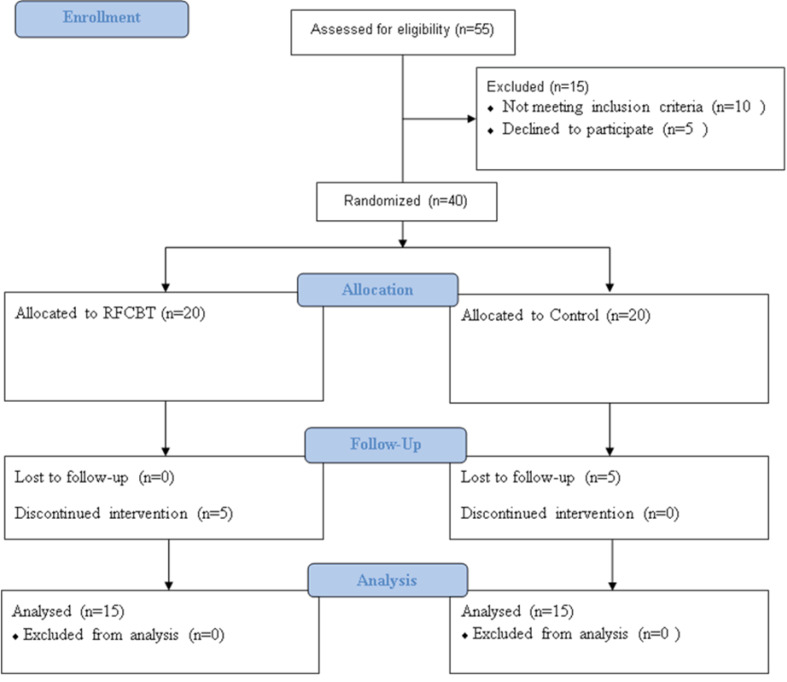
Flow diagram of study

 Fifty-five patients with CLBP were screened for eligibility (Figure-1). Ten patients didn’t meet inclusion criteria, and five patients declined to participate. Forty patients randomized and allocated in RFCBT and control groups. Five patients discontinued RFCBT during the first three sessions, and five other patients from the control group refused to participate in follow-up assessment because of pain worsening or hospitalization for other medical problems. Thirty patients from both groups participated in follow-up assessments. Post-test and follow up assessments were administered 3 and 6 months later, respectively.

 Demographic characteristics of participants in both RFCBT and control groups are depicted in the Table-2. Chi-square test showed no significant difference between RFCBT and control conditions in age (χ2=0.565, P=0.901), education (χ2=0.202, P=0.914), and sex (χ2=0.159, P=0.69) of participants.

Table-3 shows that the means of depression, anxiety, and pain severity in the RFCBT group were decreased comparing pre-treatment with post-treatment and follow-up. Each of these groups included 15 participants who were assessed during pre-treatment, post-treatment, and follow-up stages. The reported results of other statistical analyses are based on the same groups and the same number of patients.

 A mixed analysis of variance was used to determine the effect of RFCBT on depression, anxiety and pain severity in pre-treatment, post-treatment, and follow-up. Data were tested to meet mixed ANOVA assumptions. Independence assumption was met because the answers of participants were not affected by other participants. Shapiro-Wilk test was used to test the normality assumption. Distribution of scores were normal in both RFCBT and control groups for depression (Z=0.943, P=0.42; Z=0.948, P=0.494), anxiety (Z=0.931, P=0.28; Z=0.921, P=0.202), and pain severity (Z=0.879, P=0.057; Z=0.933, P=0.3). Homogeneity of variances and sphericity were checked by Levene’s test and Mauchly’s sphericity test, respectively. Homogeneity of variances assumption was met for depression (F=28, P=0.746), anxiety (F=28, P=0.781), and pain severity (F=28, P=0.753), but Mauchly’s sphericity test showed that sphericity assumption was not met for depression (Mauchly’s W=0.568, P=0.001), (Mauchly’s W=0.763, P=0.002), and pain severity (Mauchly’s W=0.62, P=0.002). Since the sphericity criterion could not be met, Greenhouse-Geisser correction was used.

 Mixed analysis of variance showed that there was a significant difference between the mean scores of depression in the pre-test, post-test, and follow-up stages (F1.17=90.85, P=0.001, η2=0.764). Bonferroni test demonstrated that depression scores in post-test (P=0.001) and follow-up (P=0.001) were significantly reduced comparing pre-test. Furthermore, the mean scores of depression in the pre-test, post-test, and follow-up assessments were significantly different in RFCBT and control groups (F1.17=60.42, P=0.001, η2=0.68). The calculated F for between-group factor was significant (F1=23.01, P=0.001, η2=0.45), which illustrated that the mean of depression scores was significantly different in RFCBT and control groups (Table-4).

 Mixed analysis of variance showed a significant difference between the mean scores of anxiety in the pre-test, post-test and follow-up assessment (F1.79=58.66, P=0.001, η2=0.67). Bonferroni demonstrated that anxiety scores in post-test (P=0.001) and follow-up (P=0.001) were significantly decreased comparing pre-test. Furthermore, the mean scores of anxiety in the pre-test, post-test, and follow-up were significantly different in RFCBT and control groups (F1.79=52.59, P=0.001, η2=0.65). The calculated F for between-group factor was significant (F1=25.7, P=0.001, η2=0.47), which showed the mean of anxiety scores was significantly different in RFCBT and control groups (Table-4).

 Also, there was a significant difference between the mean scores of pain severity in the pre-test, post-test and follow-up assessments (F1.27=38.81, P=0.001, η2=0.58). Bonferroni test showed that pain severity scores in post-test (P=0.001) and follow-up (P=0.001) were significantly decreased comparing pre-test. Furthermore, the mean scores of pain severity in the pre-test, post-test, and follow-up were significantly different in RFCBT and control groups (F1.27=29.17, P=0.001, η2=0.51). The calculated F for between-group factor was significant (F1=7.17, P=0.012, η2=0.20) indicated that the mean of pain severity scores was significantly different in RFCBT and control groups (Table-4). MANOVA showed that there was one significant effect of RFCBT on pain severity after removing the pre-test effect. This significant effect depicted a significant difference between RFCBT and control groups in at least one of the CPG components (Wilks’ lambda=0.41, F=71.10, P=0.001). One-way analysis of covariance for CPG components was used to determine this component. Table-5 shows the significant difference between RFCBT and control groups in pain intensity and pain-related disability after removing the pre-test effect. The significance level for pain intensity and pain-related disability was smaller than the significance level from Bonferroni correction (P<0.017), which was resulted from dividing 0.05 by three dependent variables.

## Discussion

This study aimed to determine the effectiveness of RFCBT on depression, anxiety, and pain severity in individuals with CLBP. The findings showed that RFCBT was effective for reducing depression, anxiety, and pain severity in patients with CLBP compared with the control group. Pain intensity and pain-related disability as two components of CPG were significantly reduced in the RFCBT group compared with the control group. Furthermore, comparing the mean scores of depression, anxiety, and pain severity in post-test and follow-up showed no significant difference, which indicates the persistence of the therapeutic benefits after three months. The effectiveness of RFCBT for depression and anxiety was consistent with the findings of Watkins et al. [13], which showed that RFCBT significantly reduced residual depression and comorbid anxiety compared with treatment as usual. Our findings of the effectiveness of RFCBT for depression and anxiety are also consistent with the findings of other studies that showed the effectiveness of group RFCBT [14] and web-based RFCBT [15] for depression and anxiety. Although the findings of these studies are not about depression and anxiety in chronic pain, the rumination as a transdiagnostic construct [21] can be associated with depression and anxiety regardless of their comorbidity with chronic pain. The effectiveness of RFCBT for depression and anxiety in patients with CLBP can be defined as the consequence of targeting this transdiagnostic construct that is the key component of the pain catastrophizing [9]. While the association of pain catastrophizing with different types of dysfunction in chronic pain, including increased rates of depression and anxiety [5,6], and the effectiveness of therapies focusing on pain catastrophizing [8] are illustrated in previous studies. Our findings also showed that RFCBT reduced pain intensity and pain-related disability in patients with CLBP as two components of pain severity. These findings are consistent with the findings of Van Damme et al. [11] and Ogunlana et al. [10], which showed the association of rumination with pain intensity and disability, respectively. The effectiveness of RFCBT for pain intensity and pain-related disability can be defined as the result of targeting rumination that is the most important factor of pain catastrophizing [9]; however, the association of pain catastrophizing with pain intensity and disability are shown in previous studies [5,6]. We found that RFCBT did not reduce pain persistence as the third component of pain severity. This finding is consistent with the findings of Von Korff et al. [20], which showed that pain intensity and pain disability are more related to psychological conditions while pain persistence provides additional useful information. The results of the study also showed the persistence of the therapeutic benefits of RFCBT after three months. The longer-term improvements and relapse prevention in RFCBT are not widely studied before. However, previous studies illustrated that the outcome and the persistence of some of the most successful psychological interventions for CLBP are mediated by reducing pain catastrophizing [22]. Focusing on the rumination as the most important component of this construct in RFCBT can define the persistence of therapeutic benefits after three months. This study has several limitations include small group size, lack of blindness, and convenience sampling which may affect the results of the study.

## Conclusion

 Our findings indicate that RFCBT may offer benefits for treating patients with CLBP by reducing depression, anxiety, and pain severity when added to their usual pharmacological treatment. This psychotherapy can be considered as a high potential treatment for chronic pain. Comparing the effectiveness of RFCBT and another psychological treatment for CLBP and longer-term follow-up may improve the quality of future researches.

## Acknowledgment

 This article has been extracted from the written thesis for a Ph.D. degree in the School of Medicine, Shahid Beheshti University of Medical Sciences (Registration No:M394). We thank the physicians and staff of shahid Modarres Hospital for their generous cooperation.

## Conflict of Interest

 The authors have no conflicts of interest in writing this article.

**Table 1:** The Details of the Sessions of Rumination-focused Cognitive-behavioral Therapy Protocol

**Session**	**Content**
**1&2**	Initial assessment, Familiarizing patients with Chronic low back pain/ The relationship between depression, rumination and chronic pain/ The impact of depression and rumination on the onset and recurrence of symptoms, Rumination, and avoidance/ Treatment and treatment logic
**3**	Checking rumination episodes recording form, Initial case formulation using Functional analysis (FA), Using Antecedent-Behavior-Consequence form, Goal setting
**4**	FA of the rumination and avoidance (antecedents, usefulness, early warning signs and alternative responses of rumination)
**5&6**	Changing the style of thinking, representing different thinking styles, if-the plans, and consolidation of these plans
**7**	Finding functional alternatives for rumination, Replacing avoidance behaviors with approaching behaviors
**8**	Changing the processing style, Explaining the difference between concrete and abstract thinking style, determination the thinking style of the patients, identifying “Why” and “How” questions, Concreteness training, and imagery
**9**	Changing the processing style, Explanation the logic of the absorption, introducing absorbing activities
**10**	Introducing self-compassionateness and the examples of being compassionate to self and others
**11&12**	Preparing patients for the treatment termination, Relapse prevention and administrating the questionnaires

**Table 2:** Demographic Characteristics of Participants

**Characteristics**		**RFCBT group** **n( %) **	**Control group** **n( %)**	**χ** ^2^	**P-value**
**Age,y**	20-30 31-40 41-50 51-55	2 (6.6) 3 (10) 5 (16.6) 5 (16.6)	3 (10) 4 (13.3) 4 (13.3) 4 (13.3)	0.565	0.901
**Education level**	High school Bachelor Master	4 (13.3) 8 (23.6) 3 (10)	3 (10) 9 (30) 3 (10)	0.202	0.914
**Sex**	Female Male	11 (36.6) 4 (13.3)	10 (33.3) 5 (16.6)	0.159	0.69

**Table 3:** Means and Standard Deviations of Variables in Pre-treatment, Post-treatment, and Follow-up Assessments

**Conditions**	**Variables**	**Pre-treatment** **Mean (SD)**	**Post-treatment** **Mean (SD)**	**Follow up** **Mean (SD)**
**RFCBT** **Control**	Depression	17.47 (1.81) 16.67 (1.68)	9.07 (3.65) 15.60 (2.59)	8.93 (3.75) 16.01 (2.24)
**RFCBT** **Control**	Anxiety	13.53 (2) 12.73 (1.94)	7.60 (1.96) 12.67 (1.80)	7.93 (1.67) 12.47 (1.96)
**RFCBT** **Control**	Pain severity	38.80 (2.91) 37.47 (3.16)	32.20 (2.70) 36.87 (3.87)	32.27 (3.15) 37.13 (3)

**Table 4:** Mixed Analysis of Variance for Depression, Anxiety, and Pain Severity Scores with Greenhouse-Geisser Correction

**Variables**		**Statistical indices**	**SS**	**df**	**MS**	**F**	**P-value**	**Effect size**	**Power**
**Depression**	Within-Group	Test (Repeating Measurement ) Test*Group Error	435.82 289.86 134.31	1.17 1.17 32.78	372.17 247.53 4.09	90.85 60.42	0.001 0.001	0.764 0.68	0.99 0.99
	Between-Group	Group Error	409.6 498.22	1 28	409.6 17.79	23.01	0.001	0.45	0.99
**Anxiety**	Within-Group	Test (Repeating Measurement ) Test*Group Error	176.08 157.86 84.4	1.79 1.79 50.24	98.12 87.96 1.67	58.66 52.59	0.001 0.001	0.67 0.65	0.99 0.99
	Between-Group	Group Error	193.6 216.22	1 28	193.60 7.72	25.7	0.001	0.47	0.99
**Pain severity**	Within-Group	Test (Repeating Measurement ) Test*Group Error	247.75 186.2 178.71	1.27 1.27 35.66	194.53 146.2 5.01	38.81 29.17	0.001 0.001	0.58 0.51	0.99 0.99
	Between-Group	Group Error	168.1 656.22	1 28	168.1 23.43	7.17	0.012	0.2	0.98

**Table 5:** One-Way Analysis of Covariance for CPG Components

**Dimensions** **of variable**	**Source ** **of variation**	**SS**	**Df**	**MS**	**F**	**P-value**	**Eta**
**Pain intensity**	Group Error	53.12 71.07	1 25	53.12 2.84	18.69	0.00	0.43
**Pain persistence **	Group Error	1.87 66.82	1 25	1.87 2.67	0.7	0.41	0.03
**Pain-related** **disability**	Group Error	17.65 34.93	1 25	17.65 1.4	12.64	0.00	0.34
